# Aerobic Exercise Improves Cognitive Functioning in People With Schizophrenia: A Systematic Review and Meta-Analysis

**DOI:** 10.1093/schbul/sbw115

**Published:** 2016-08-12

**Authors:** Joseph Firth, Brendon Stubbs, Simon Rosenbaum, Davy Vancampfort, Berend Malchow, Felipe Schuch, Rebecca Elliott, Keith H. Nuechterlein, Alison R. Yung

**Affiliations:** 1 Institute of Brain, Behaviour and Mental Health, University of Manchester, Manchester, UK;; 2 Physiotherapy Department, South London and Maudsley NHS Foundation Trust, London, UK;; 3 Health Service and Population Research Department, Institute of Psychiatry, Psychology and Neuroscience, King’s College London, London, UK;; 4 Department of Exercise Physiology, School of Medical Sciences, Faculty of Medicine, University of New South Wales, Sydney, New South Wales, Australia;; 5 KU Leuven Department of Rehabilitation Sciences, Leuven, Belgium;; 6 KU Leuven Department of Neurosciences, UPC KU Leuven, Leuven, Belgium;; 7 Department of Psychiatry and Psychotherapy, Ludwig-Maximilians-University, Munich, Germany;; 8 Hospital de Clínicas de Porto Alegre, Porto Alegre, Brazil;; 9 School of Psychological Sciences, University of Manchester, Manchester, UK;; 10 Department of Psychiatry and Biobehavioral Sciences, David Geffen School of Medicine at UCLA, Los Angeles, CA;; 11 Department of Psychology, University of California, Los Angeles, Los Angeles, CA;; 12 Greater Manchester West NHS Mental Health Foundation Trust, Manchester, UK; 13 These joint-first authors contributed equally to the writing of this manuscript.

**Keywords:** physical activity, cognition, neurocognitive, neurocognition, psychosis

## Abstract

Cognitive deficits are pervasive among people with schizophrenia and treatment options are limited. There has been an increased interest in the neurocognitive benefits of exercise, but a comprehensive evaluation of studies to date is lacking. We therefore conducted a meta-analysis of all controlled trials investigating the cognitive outcomes of exercise interventions in schizophrenia. Studies were identified from a systematic search across major electronic databases from inception to April 2016. Meta-analyses were used to calculate pooled effect sizes (Hedges g) and 95% CIs. We identified 10 eligible trials with cognitive outcome data for 385 patients with schizophrenia. Exercise significantly improved global cognition (*g* = 0.33, 95% CI = 0.13–0.53, *P* = .001) with no statistical heterogeneity (*I*^2^ = 0%). The effect size in the 7 studies which were randomized controlled trials was *g* = 0.43 (*P* < .001). Meta-regression analyses indicated that greater amounts of exercise are associated with larger improvements in global cognition (β = .005, *P* = .065). Interventions which were supervised by physical activity professionals were also more effective (*g* = 0.47, *P* < .001). Exercise significantly improved the cognitive domains of working memory (*g* = 0.39, *P* = .024, *N* = 7, *n* = 282), social cognition (*g* = 0.71, *P* = .002, *N* = 3, *n* = 81), and attention/vigilance (*g* = 0.66, *P* = .005, *N* = 3, *n* = 104). Effects on processing speed, verbal memory, visual memory and reasoning and problem solving were not significant. This meta-analysis provides evidence that exercise can improve cognitive functioning among people with schizophrenia, particularly from interventions using higher dosages of exercise. Given the challenges in improving cognition, and the wider health benefits of exercise, a greater focus on providing supervised exercise to people with schizophrenia is needed.

## Introduction

Schizophrenia is associated with impairments in cognitive functioning. ^1^ Deficits of 1–2 SDs below the general population are evident in various domains of cognition from the onset of illness and persist over time. ^2^ These cognitive impairments contribute significantly to the poor functional outcomes and long-term disability often observed among patients. ^1^ Antipsychotic medications have little impact on improving cognition, ^3^ and other pharmacological approaches towards treating cognitive deficits have demonstrated limited efficacy thus far. ^4^

Nonpharmacological interventions have been developed to specifically target cognitive symptoms, including cognitive remediation therapy (CRT). CRT involves completing tasks designed to train various cognitive functions such as memory, attention, and problem-solving skills. A meta-analysis of 40 studies with 2104 participants found that CRT improves cognitive functioning significantly more than control conditions, with effect sizes within the moderate range. ^5^ However, CRT has only a small effect on psychiatric symptoms, and improvements are lost over time. ^5^

Novel nonpharmacological strategies that can improve cognition, symptoms, and socio-occupational functioning would provide valuable adjunctive treatments for schizophrenia. A number of recent meta-analyses have shown that physical activity, and particularly structured exercise, can significantly improve positive symptoms, negative symptoms, and social functioning in this population. ^6–8^ Furthermore, by increasing cardiorespiratory fitness and metabolic health, exercise may also reduce the physical health problems associated with schizophrenia, such as obesity and diabetes, which contribute towards reduced life expectancy. ^9^

In the general population, exercise has been shown to have modest effects on attention, processing speed, memory, and executive functioning, ^10^ perhaps through stimulating neuroplasticity. ^11^ Exercise has also been found to increase hippocampal volume and white matter integrity in healthy older adults ^12,13^ and those with schizophrenia. ^14,15^ Additionally, cross-sectional research in people with schizophrenia has demonstrated that physical activity and fitness are associated with better cognitive performance, ^16^ greater grey and white matter volumes, ^17^ and higher levels of neurotrophic factors which promote brain plasticity. ^18^ A number of narrative reviews have also discussed the potential benefits of exercise on brain health and cognition. ^19–21^ However, earlier meta-analyses of exercise in schizophrenia have not been able to determine the effects on cognition due to insufficient data (including only 2–4 aerobic exercise trials for each cognitive domain), ^8^ although many additional studies have since been published.

The aim of this meta-analysis was to assess the effect of exercise on global cognition in people with schizophrenia, along with examining which domains of cognitive functioning are most sensitive to exercise interventions. We also sought to explore the impact of various patient and intervention characteristics which affect the outcomes of exercise interventions, using meta-regression analyses.

## Methods

This meta-analysis followed the PRISMA statement ^22^ to ensure comprehensive and transparent reporting of methods and results.

### Search Strategy

Two independent authors (J.F. and B.S.) conducted an electronic database search of Cochrane Central Register of Controlled Trials, Health Technology Assessment Database, AMED (Allied and Complementary Medicine), HMIC Health Management Information Consortium, Ovid MEDLINE, PsycINFO, Embase from inception to April 2016. The search terms used were: “schizo*” or “psychosis” or “psychotic” and “exercise” or “physical activity” or “fitness” or “aerobic” or “resistance training” and “neuro*” or “cogniti*”. A search of Google Scholar was conducted using the same key words to identify any additional relevant articles. The reference lists of retrieved articles were also searched. Only English-language research articles were included in the review.

### Eligibility Criteria

We aimed to include all published studies examining the neurocognitive outcomes of exercise interventions for people with schizophrenia, in comparison to a control condition. Eligible populations included any sample in which the majority of patients were being treated for schizophrenia or schizoaffective disorder. Specifically, studies which included a broad spectrum of psychiatric disorders were only included if >80% of the sample had a clinically diagnosed nonaffective psychotic disorder. Data from studies in which <80% of the sample had a nonaffective psychotic disorder were only eligible if the outcome data specifically for the schizophrenia/nonaffective psychosis subgroup could be accurately determined.

For the purpose of this review, exercise was defined as structured and repetitive physical activity that has an objective of improving or maintaining physical fitness. ^23^ Interventions using only yoga or tai-chi were excluded from the analyses, as these theoretically confer benefits for cognition which are distinct from the physical activity itself. ^24^ Studies which implemented physical activity as an active-control condition for cognitive training interventions were not used in the analyses (unless a passive control arm was also available for comparisons with exercise). Interventions which combined exercise with other psychosocial or pharmacological treatments were only included if the nonexercise aspects of the intervention were controlled for in the comparison conditions.

### Data Extraction

Articles were independently screened by 2 reviewers (J.F. and B.S.) to assess eligibility. Disagreements were resolved through discussion until consensus was reached. A systematic tool was used to extract all of the following data from each study:

1. Primary outcome—global cognition: This was defined as average change in all clinically validated measures of cognitive functioning following an exercise intervention (or control condition). Where changes in multiple cognitive tasks/domains were reported, a composite change score was calculated from the average change in each individual task/cognitive domain. This method has been applied in previous meta-analyses examining the effects of cognitive training interventions in schizophrenia. ^5,25^ All neurocognitive tasks used to calculate these composite scores are shown in supplementary material 1.
2. Secondary outcomes—individual cognitive domains: Effects of exercise in individual cognitive domains were examined with respect to the categories established by the NIMH-MATRICS Neurocognition Committee, based on factor analytic studies of the structure of cognition in schizophrenia, ^26^ and subsequently used to guide the structure of the MATRICS Consensus Cognitive Battery (MCCB). ^27^ This specifies 7 individual domains of cognition which are: speed of processing, attention/vigilance, working memory, verbal learning and memory, visual learning and memory, reasoning and problem solving, and social cognition. Where studies had not used the gold-standard MCCB itself, the tasks used were categorized into their respective domains, ^26^ as in previous meta-analyses. ^5,25^ supplementary material 1 displays the categorization of all cognitive tasks used in meta-analyses.
3. Potential moderators: Data on factors which may influence the effect size of exercise interventions were also extracted from each study, including sample characteristics (age, gender distribution, years of illness duration), exercise intervention characteristics (minutes of exercise per week, length of intervention in weeks, improvements in fitness/maximal oxygen uptake, professional background of instructor), and study design (control condition used and trial quality).

Where unreported study data were required to determine eligibility or for meta-analyses, the corresponding authors of respective studies were contacted up to 2 times to request the variables of interest.

### Statistical Analyses

All analyses were performed in Comprehensive Meta-Analysis 2.0, ^28^ with a random-effects model applied throughout to account for the expected heterogeneity between studies. ^29^ Pooled effect sizes for exercise on both global cognition and each individual cognitive domain were calculated as Hedges’ *g* using the mean change scores (and SDs) in exercise and control conditions. Where raw means were not reported, the effect size was computed from *F*-statistics or *t* values. The heterogeneity between studies was quantified using Cochran’s *Q* and *I*^2^ values, which estimate the amount of heterogeneity resulting from between-study variance, rather than by chance. The risk of bias for each study was examined using the Cochrane’s Collaboration risk of bias tool ^30^ which assesses 6 aspects of trial methodology (sequence generation, allocation sequence concealment, blinding of participants and personnel, blinding of outcome assessment, incomplete outcome data, and selective outcome reporting) that could potentially introduce different sources of bias. To account for publication bias, the Eggers’ test was applied and the Fail-Safe Number ^31^ was calculated to determine the number of unpublished null studies which would be required to invalidate our findings (*P* > .05). We also performed sensitivity analyses to assess if comparable effects were still observed following the removal of low-quality or nonrandomized controlled trials.

The relationship between continuous moderators and effect size estimates were explored with meta-regression analyses. These were performed for putative patient and treatment characteristics which may impact upon the cognitive outcomes of exercise in schizophrenia. Patient moderators included age, gender, and duration of illness. Treatment moderators were intervention length, weekly amounts of exercise, and improvements in cardiorespiratory fitness. The impact of categorical moderators of exercise supervision and intervention content were assessed using subgroup analyses.

## Results

### Search Results

**Fig. 1.**
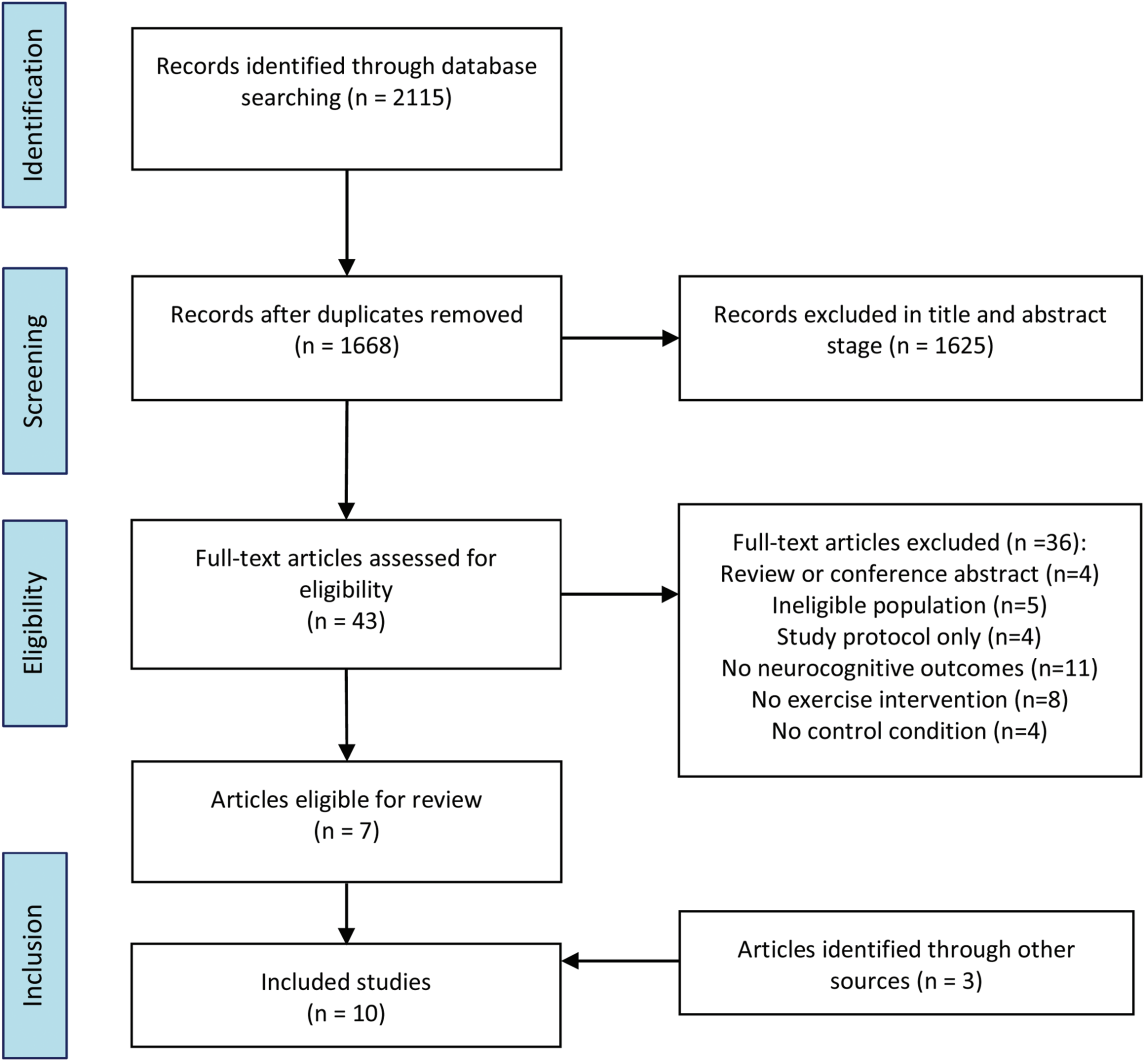
PRISMA flow diagram of systematic search and study selection.

The initial database search was performed on April 9, 2016. The search returned 2115 results reduced to 1668 after duplicates were removed. A further 1625 articles were excluded after reviewing the titles and abstracts for eligibility. Full text versions were retrieved for 43 articles, of which 7 were eligible for inclusion. Reasons for exclusion and full details of the search results are summarized in figure 1. A further 2 articles were identified from a similar search of Google Scholar, and one “in-press” publication was retrieved from its corresponding author. A total of 10 articles, each with unique samples, were eligible for inclusion. Additional data for 3 studies was obtained from the corresponding authors. ^32–34^

### Included Studies and Participant Details

Study characteristics and intervention details are displayed in table 1. Two studies were conducted in the United States, 2 in Germany, 2 in China, and 1 each in Brazil, Portugal, Netherlands, and India. There was a total of 592 psychiatric patients across the studies: 221 were assigned to exercise, 234 to control conditions, and 137 to ineligible study arms including tai-chi ^35^ and yoga. ^36,37^ In the eligible samples, 92.1% had schizophrenia/schizoaffective disorder and 7.9% had other nonaffective psychotic disorders. The mean age was 37.3 years (range = 22.7–55 y), mean duration of illness was 13.4 years (9.2 mo to 30.7 y) and 56% were male.

**Table 1.** Details of Included Studies

		Sample Characteristics								Exercise Intervention								Study Details		
		Exercise (*n*)		Control (*n*)		Mean Age		% Male		Session Content		Supervision		Weeks + Sessions		Comparator		Cognitive Domains Examined		Risk of Bias^a^
Behere et al (2011) ^37 ^		17		22		31.8		76.2		60-min “National Fitness Corps” program, consisting of brisk walking, jogging, and exercises in standing and sitting postures.		Yoga instructor		12-wk sessions per week: NS		Waitlist (randomized)		Social cognition		Low
Campos et al (2015) ^39 ^		13		16		39.4		72.8		20min of an interactive physical activity videogame “Move4Health.” This demands upper and lower limb movements in various grape-related games. Intensity and difficulty increases overtime.		Rehabilitation center staff		8wk, 2 per week		TAU (nonrandomized)		Processing speed		High
Ho et al (2016) ^35 ^		51		49		54.9		50.0		60min designed to match physical exertion of tai-chi (50%–60% maximal oxygen consumption). Consists of stretching and joint movements, walking, stepping, mild weight training, and cool-down.		Mental health professionals		12wk, 3 per week		Waitlist (randomized)		**Working memory** ^**b**^		Low
Kimhy et al (2015) ^32 ^		13		13		36.9		64		60min of mixed aerobic exercise at 60%–75% VO_2_ peak. Sessions contain a mixture of treadmill running, elliptical training, and interactive video games.		Physical trainer		12wk, 3 per week		TAU (randomized)		Processing speed		Low
																		**Attention/vigilance**		
																		Working memory		
																		Verbal learning		
																		Visual learning		
																		Reasoning		
																		**Social cognition**		
Lin et al (2015) ^36 ^		31		33		24.9		0.0		45–60min of aerobic exercise at 50%–60% VO_2_ max. Warm up, treadmill, stationary cycling, followed by cool-down.		Yoga instructor		12wk, 3 per week		TAU (randomized)		Processing speed^b^		Low
																		Attention/vigilance^b^		
																		**Working memory** ^b^		
																		**Verbal learning** ^b^		
Malchow et al (2015) ^34 ^		22		21		36.5		72.0		30min of stationary cycling at an individually defined intensity (based on blood lactate concentrations) that was gradually increased over the intervention.		Sports scientist		12wk, 3 per week		Table football + CR (nonrandomized)		Processing speed		High
																		Working memory		
																		Verbal learning		
																		Reasoning		
Nuechterlein et al (2016) ^33 ^		7		9		22.7		73.0		30–45min of aerobic work-out video at 60%–80% of max. heart rate. Workout videos included calisthenics (eg, lunges, squats, pushups) and simple movement sequences at varying levels of intensity.		Physical trainer		10wk, 4 per week		CR (nonrandomized)		Processing speed^b^		High
																		Attention/vigilance		
																		Working memory^b^		
																		Verbal learning^b^		
																		Visual learning^b^		
																		Reasoning^b^		
																		**Social cognition** ^**b**^		
Oertel-Knöchel et al (2014) ^38 ^		8		11		42.3		44.4		45-min workout with warm-up (10min), followed by circuit training (25min) using trampolines, weights, physiotherapy balls, staves, and flexi bars at 60%–70% of max. heart rate and ending with a cool-down phase (10min).		Physical trainer		4wk, 3 per week		Relaxation training + CR (randomized)		**Processing speed** ^b^		Low
																		**Working memory** ^b^		
																		Verbal learning^b^		
																		**Visual learning** ^b^		
Pajonk et al (2010) ^14 ^		8		8		35		100		30min of stationary cycling at an individually defined intensity (based on blood lactate concentrations) that was gradually increased over the intervention.		Study investigator		12wk, 3 per week		Table football (randomized)		Working memory		Low
																		**Verbal learning**		
Svatkova et al (2015) ^15 ^		16		17		30.1		81.7		40min of aerobic training (cycling, treadmill, elliptical) at up to 75% of max. heart rate followed by 20min of resistance training.		Physical trainer		24wk, 2 per week		Occupational therapy (randomized)		Global only (IQ)		Low

*Note*: Bold indicates statistically significant improvement in exercise group compared to control condition for cognitive subdomain. CR, cognitive remediation; IQ, intelligence quotient; NS, nonsignificant; TAU, treatment as usual.

^a^Assessed with “Cochrane Risk of Bias” tool.

^b^Intention-to-treat data available for cognitive domain analysis.

Intervention details are shown in table 1. Exercise programs were, on average, 12.2 weeks long (range = 4–24wk) with 2.9 sessions per week (range = 2–4 sessions) of 20–60 minutes in duration. All primarily focused on aerobic exercise, although 3 also incorporated resistance-based (muscle strengthening) training. ^15,35,38^ The common modalities were aerobic machines such as cycle ergometers/treadmills (*N* = 5), bodyweight exercises (*N* = 3), interactive video games (*N* = 2), and free-weights (*N* = 2). Three interventions combined exercise with cognitive remediation (CR) but were still included in the analyses, since their comparison conditions controlled for this using CR alone, ^33^ CR with table football, ^34^ or CR with relaxation training. ^38^ Other control conditions were table football alone (*N* = 1), occupational therapy (*N* = 1), and treatment-as-usual (*N* = 5). Risk of bias assessments identified 3 nonrandomized studies with high risk of bias influencing results. ^33,34,39^ The 7 studies which were randomized controlled trials (RCTs) rated “low risk of bias” for most items, although 1 had used nonblinded outcome assessments and 2 were at risk of bias from incomplete outcome data with no intention-to-treat (ITT) analyses. Bias assessment results for individual studies are displayed in supplementary material 2.

### Meta-Analysis of the Neurocognitive Outcomes of Exercise

**Fig. 2.**
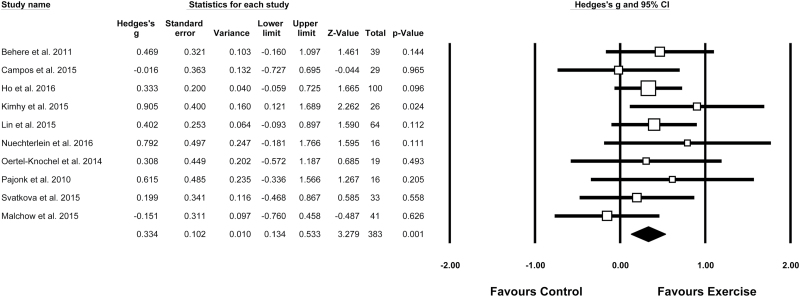
Meta-analysis of exercise effects on global cognition in comparison to control conditions. Box size represents study weighting. Diamond represents overall effect size and 95% CIs.

Outcome data were available for 186 participants in the exercise group and 199 in control groups. The effect of exercise on global cognition is displayed in figure 2 (*N* = 10, *n* = 383). This shows exercise interventions significantly improved overall cognitive performance (*g* = 0.33, 95% CI = 0.13–0.53, *P* = .001). There was no evidence of statistical heterogeneity in the pooled effect size (*Q* = 7.0, *P* = .64, *I*^2^ = 0%). Egger’s regression test showed no evidence of publication bias (*t* = 0.786, *P* = .454). The fail-safe *N* was 20, indicating that 20 additional “null” studies would be needed for the observed *P* value to exceed .05.

A sensitivity analysis was performed on the RCTs only while also excluding studies with high risk of bias (see table 2). Among the RCTs (*N* = 7, *n* = 297), the effect size was *g* = 0.41 (95% CI = 0.19–0.64, *P* < .001) with low heterogeneity between studies (*Q* = 2.32, *P* = .88, *I*^2^ = 0%).

**Table 2.** Cognitive Outcomes of Exercise Interventions in People With Schizophrenia

			Meta-Analysis				Heterogeneity		
	Studies	Total *n*	Hedge’s *g*	95% CI		*P* Value	*Q* Value	*P* Value	*I* ^2^%
Global cognition									
Overall	10	383	0.334	0.13	0.53	**.001**	7.00	.64	0
RCTs only	7	297	0.412	0.19	0.64	**<.001**	2.32	.88	0
Exercise instructor	6	197	0.466	0.19	0.75	**<.001**	2.44	.79	0
Other instructor	4	186	0.196	−0.09	0.48	.178	2.81	.42	0
Exercise alone vs standard control	7	307	0.377	0.15	0.60	**.001**	3.56	.74	0
Exercise + CRT vs CRT control	3	76	0.209	−0.33	0.75	.447	2.73	.23	26.8
Cognitive domains									
Working memory	7	282	0.390	0.05	0.73	**.024**	10.9	.09	45.1
Processing speed	6	195	0.125	−0.15	0.40	.375	4.97	.42	0
Verbal learning and memory	6	166	0.284	−0.09	0.64	.138	7.76	.17	35.6
Reasoning and problem solving	4	146	−0.100	−0.42	0.22	.528	1.36	.72	0
Attention/vigilance	3	104	0.663	0.20	1.12	**.005**	2.51	.07	20.3
Social cognition	3	81	0.712	0.27	1.15	**.002**	1.23	.54	0
Visual learning and memory	3	61	0.004	−0.45	0.52	.889	0.67	.71	0

*Note*: Bold values represent a statistically significant difference between exercise and control conditions. CRT, cognitive remediation therapy; RCT, randomized controlled trial.

### Factors Associated With Intervention Effectiveness

All exercise interventions were delivered in a supervised setting. Therefore, subgroup analyses were performed to examine interventions that were supervised by a physical activity professional versus those which were not (table 2). Exercise interventions supervised by physical activity professionals (including physical trainers and yoga teachers) significantly improved global cognition (*N* = 6, *n* = 197, *g* = 0.47, 95% CI = 0.19–0.75, *P* < .001, *Q* = 2.44, *I*^2^ = 0%) whereas those supervised by other professionals (ie, mental health support and research staff) did not (*N* = 4, *n* = 186, *g* = 0.2, 95% CI = −0.09 to 0.48, *P* = .5, *Q* = 1.81, *I*^2^ = 0%), although the difference between subgroups was not significant (*P* = .19). Exercise as a stand-alone intervention was compared to treatment as usual or time- and attention-matched control conditions in 7 trials and was found to significantly improve global cognition (*N* = 7, *n* = 307, *g* = 0.38, 95% CI = 0.15–0.60, *P* < .001, *Q* = 3.56, *I*^2^ = 0%). Three trials (total *n* = 76) examined the additional benefits of exercise as an add-on to CRT, in comparison to CRT as a control condition. Random-effects meta-analysis found no significant differences between exercise plus CRT vs CRT control conditions (*g* = 0.21, 95% CI = −0.33 to 0.75, *P* = .45, *Q* = 2.73, *I*^2^ = 26.8).

Meta-regression analyses suggested that a greater amount of exercise (in minutes per week) was associated with a larger improvement in global cognition (supplementary material 3), which closely approached statistical significance (*N* = 9, *n* = 344, *B* = 0.0054, SE = 0.0029, *Z* = 1.85, *P* = .065). “Improvements in fitness” had a nonsignificant tendency to correlate with intervention effect size (*N* = 7, *n* = 222, *B* = 0.61, SE = 0.352, Z = 1.74, *P* = .082). None of the other intervention factors (length in weeks, sessions per week) or sample characteristics (age, duration of illness, % male) were associated with effects on cognitive performance (all *P* ≥ .1).

### Effects of Exercise on Individual Cognitive Domains

We also examined the effects of exercise in the 7 individual cognitive domains established by the MATRICS Neurocognition Committee ^26,27^ (supplementary material 1). Effects across all domains are displayed in table 2. The most widely assessed was “working memory” (*N* = 7, *n* = 282), within which exercise resulted in significant improvements vs control conditions (*g* = 0.39, 95% CI = 0.05–0.73, *P* = .024) with medium heterogeneity (*Q* = 10.92, *P* = .091, *I*^2^ = 45.1%). “Processing speed” and “verbal learning and memory” were each assessed by 6 studies (*n* = 195, *n* = 166) but showed no significant difference from control conditions (processing speed: *g* = 0.13, 95% CI = −0.15 to 0.40, *P* = .38, *I*^2^ = 0%; verbal learning: *g* = 0.28, 95% CI = −0.09 to 0.64, *P* = .14, *I*^2^ = 35.6%). “Reasoning and problem solving,” assessed in 4 studies also showed no benefits from exercise (*g* = −0.10, 95% CI = −0.42 to 0.22, *P* = .53, *n* = 146, *I*^2^ = 0%). The remaining 3 domains were assessed by 3 studies each. Significant effects of exercise were observed in tasks of “social cognition” (*g* = 0.71, 95% CI = 0.27–1.15, *P* = .002, *n* = 81, *I*^2^ = 0%) and “attention/vigilance” (*g* = 0.66, 95% CI = 0.20–1.12, *P* = .005, *n* = 104, *I*^2^ = 20.3%), although there were no significant differences from control conditions for “visual learning and memory” (*g* = 0.004, 95% CI = −0.45 to 0.52, *P* = .889, *n* = 61, *I*^2^ = 0%).

## Discussion

This meta-analysis set out to examine the effects of exercise on cognitive functioning in people with schizophrenia. Ten studies with 385 participants were eligible; most of which used aerobic exercise interventions. Pooled effect sizes across all cognitive outcomes showed that exercise improves global cognition significantly more than control conditions (figure 2). This was a robust finding, with little statistical heterogeneity between studies and an effect size of *g* = 0.33 across all studies (95% CI = 0.13–0.53, *P* = .001). Subgroup analyses suggest that supervision from physical activity instructors results in better cognitive outcomes. Meta-regression analyses indicate that higher weekly duration of exercise tends to be associated with greater improvement in cognition (*P* = .065). Domain-specific analyses found that exercise is particularly beneficial for social cognition (*g* = 0.71), working memory (*g* = 0.39), and attention (*g* = 0.66). This also suggests aerobic exercise may be more effective for cognition in schizophrenia than yoga, which previous meta-analyses have found to only be effective for long-term memory (with a smaller effect size of *g* = 0.32). ^8^

In this meta-analysis, the effect size in RCTs was 0.43 (95% CI = 0.21–0.66), indicating that exercise has similar effects on cognition in schizophrenia to CRT, which has an average effect size of *g* = 0.45 (95% CI = 0.31–0.59) in randomized trials. ^5^ Studies in healthy populations have shown that interventions which combine aerobic exercise with cognitively demanding tasks confer maximal benefits for cognition. ^40^ Animal research suggests this is due to aerobic exercise and learning tasks having independent but complementary effects on neurogenesis; with the combination of these 2 activities leading to 30% more new neurons than either alone. ^41^ Specifically, this may be attributed to exercise stimulating cell proliferation, and learning tasks supporting the survival of new cells. ^42^ Three studies identified in this review assessed the effects of exercise plus CRT in comparison to CRT alone. ^33,34,38^ However, there was substantial heterogeneity between the exercise training programs used, and pooled effect size found no significant benefits of “CRT plus exercise” compared to CRT control conditions (*g* = 0.21, *P* = .45) for global cognitive performance. Nonetheless, individual studies have shown significantly greater improvements from combining CRT with aerobic exercise for various cognitive subdomains (including social cognition, working memory), along with significantly greater reductions in negative symptoms of schizophrenia. ^33,34^

The cellular processes through which exercise increases neurogenesis and cognitive performance is not fully understood, although evidence from human and animal studies indicates that it is related to an increase in brain-derived neurotrophic factor (BDNF). ^43^ BDNF is the most abundant growth factor in the human brain and is upregulated in response to aerobic exercise. ^44,45^ There is also some preliminary evidence supporting the role of BDNF as a mediating factor for cognitive improvements from exercise in schizophrenia. ^32,33^ However, the benefits of exercise in schizophrenia cannot be attributed to BDNF alone, given the lack of available data examining other potential mechanisms.

For instance, it is possible that positive effects on cognition occur indirectly, since exercise has previously been shown to improve psychiatric symptoms, quality of life, and social functioning in people with schizophrenia ^6–8^ —all of which are associated with neurocognition. ^46^ Physiological changes that occur in response to exercise, such as weight-loss and improved cardiorespiratory fitness, are also linked with increased cognitive performance. ^47,48^ Indeed, our meta-regression analyses revealed a nonsignificant tendency for an association between improvements in cognition and cardiorespiratory fitness, although this was underpowered to detect a significant effect (*P* = .08). Nonetheless, 3 of the studies included in this review did report significant correlations within their respective samples between increased fitness and improvements in brain structure and function. ^14,15,32^ Further exploration of the potential mechanisms through which exercise can improve cognition in schizophrenia is an important step for understanding these effects, and for informing the design and delivery of future interventions.

Only the interventions which were supervised by physical activity professionals significantly improved global cognition (*g* = 0.47, *P* < .001). This may be due to increased exercise engagement among participants or better program delivery resulting in more favorable outcomes. Exercise dosage appears to be an important factor for achieving cognitive enhancement, as previous studies have shown that the amount or dose of exercise achieved by participants during an intervention is a significant predictor of cognitive improvements. ^49,50^ In our meta-regression analyses, minutes per week of exercise closely approached significance as a moderator variable (*P* = .065). Although other dose-related variables (ie, duration [wk] of the exercise intervention and number of sessions per week) did not have any relationship with intervention effectiveness, this may be due to the lack of variation across the included studies preventing our meta-regression analyses from detecting a relationship, as most interventions were between 8 and 12 weeks long, with 2 or 3 sessions per week (table 1). Furthermore, we were unable to analyze how exercise intensity may influence outcomes due to how this variable was reported across trials. Kimhy et al ^50^ previously examined the relative influence of exercise duration, frequency, and intensity on cognitive improvements following a 12-week exercise program in schizophrenia and found that intensity was the best predictor variable. Thus, along with determining required doses in “minutes per week,” future research should also aim to establish the length, frequency, and intensity of exercise training required to improve cognition.

Among the different domains assessed (table 2), social cognition showed the greatest improvements in response to exercise (*g* = 0.71, 95% CI = 0.27–1.15, *P* = .002). Social cognitive impairments persevere from the onset of illness across the course of schizophrenia. ^51^ They are negatively associated with employment and independent living, ^52,53^ and social cognition is more strongly predictive of real-world functioning than neurocognitive performance. ^54^ Thus, the large effects in this domain are encouraging, suggesting that the cognitive benefits of exercise may generalize to improve psychosocial and occupational outcomes for people with schizophrenia.

The 2 other domains which showed significant changes in response to exercise were attention (*g* = 0.66) and working memory (*g* = 0.39). Since these factors are strong predictors of functional recovery after a first episode of schizophrenia, ^55^ implementing exercise interventions from the early stages of illness may facilitate functional recovery. Indeed, exercise may confer even greater benefits in the early psychosis, as cognitive enhancement interventions are more effective at this time than later in illness. ^56^ Consistent with this, 3 recent studies in young patients with first-episode psychosis (aged 23–26) have observed large cognitive improvements from moderate/vigorous exercise after just 10–12 weeks. ^33,36,46^ With the currently limited evidence, it is unclear whether this high level of responsiveness to exercise among first-episode patients is due to their younger age or their earlier stage of illness. It is also possible that exercise interventions could be particularly beneficial for older patients with schizophrenia, whom typically have greater health-related comorbidities (such as hypertension, obesity, and diabetes)—since these conditions adversely affect cognitive functioning, ^57,58^ and yet improve in response to exercise training. Additionally, future studies should examine how the effects of exercise are influenced by other biological variables, such age, body mass index, and genetic variation in BDNF secretion (ie, BDNF val66met polymorphism), as these have previously been shown to modulate effects of exercise on neurocognition. ^58–60^

One limitation of this meta-analysis is that several of the included studies did not use ITT analyses and failed to report outcome data for 15.8% and 10.7% of participants enrolled in the exercise and control conditions, respectively. In CRT studies, while earlier meta-analyses of smaller trials found moderate effects on cognitive functioning, 2 recent multisite RCTs with ITT analyses have found no benefits of CRT beyond control conditions. ^61,62^ Therefore, large-scale RCTs of exercise with complete outcome data (or ITT analyses) should now be conducted to establish the efficacy for improving cognition in schizophrenia. If proven effective, exercise could present a widely beneficial and cost-effective intervention for policy makers to consider for dissemination, since it has also been found to improve cardiovascular health and symptoms in schizophrenia. ^6,63^

Some cognitive subdomains were only measured in a small number of studies (*N* = 3), which limits the strength of findings for these domains. It should also be noted that the differences in the effects of exercise across specific cognitive domains could be attributable to discrepancies in cognitive measures used. This is because cognitive tasks vary in their sensitivity to detect improvement, depending on various psychometric properties such as task difficulty, reliability, and standard variance in performance.

A further consideration is that all interventions primarily used aerobic exercise. Single-arm studies (ineligible for this meta-analysis) using resistance training methods in people with early psychosis ^46^ and long-term schizophrenia ^64^ have demonstrated significant benefits for verbal memory and processing speed—domains which did not show improvement from aerobic exercise in this meta-analysis. Furthermore, a recent RCT of 20-week resistance training for schizophrenia found significant increases in BDNF. ^65^ Despite the positive effects of resistance training for cognition observed in other populations, ^66,67^ no RCTs have measured this in schizophrenia to date.

In conclusion, the available evidence indicates that exercise improves cognitive functioning in people with schizophrenia, particularly within domains of social cognition, working memory, and attention, all of which are predictive of socio-occupational outcomes. Our data suggest that supervision from physical activity professionals and higher levels of weekly exercise are important for promoting the cognitive benefits of exercise. Future research should aim to explore the mechanisms of exercise-induced cognitive improvements, determine if this is related to increased cardiorespiratory fitness, establish required dosages of exercise, and investigate the effectiveness of resistance training. Furthering current understanding in these areas will help to develop optimal programs, which may involve combining exercise training with CRT. Given the known benefits of exercise for psychiatric symptoms, social functioning, and physical health, ^6–8^ feasible and accessible methods for delivering exercise in clinical practice should be explored and implemented. ^21^

## Supplementary Material

Supplementary material is available at http://schizophreniabulletin.oxfordjournals.org.

## Funding

J.F. is funded by an MRC Doctoral Training Grant. D.V. is funded by the Research Foundation – Flanders (FWO-Vlaanderen). S.R. is funded by a Society for Mental Health Research Early Career Fellowship (Australia). K.H.N. is funded by NIMH, Janssen, Stanley Medical Research Institute, and Posit Science. B.M. is funded by the German Federal Ministry of Education and Research (BMBF: 01EE1407AE).

## Supplementary Material

Supplement_1._Neurocognitive_tasks

Supplement_2._Risk_of_bias_assessments

Supplement_3._Meta_regression
