# Supplementary material for: Aerobic Exercise Improves Cognitive Functioning in People With Schizophrenia: A Systematic Review and Meta-Analysis
Source: Schizophr Bull. 2016 Aug 12;43(3):546–56. doi: 10.1093/schbul/sbw115 (PMC5464163; doi:10.1093/schbul/sbw115)
Supplement: Supplement_1._Neurocognitive_tasks [file sbw115_suppl_Supplement_1._Neurocognitive_tasks.doc]

| **Supplement 1.** Categorization of neurocognitive tasks used in the meta-analysis^a^ | |
| --- | --- |
| Cognitive Domain | Neurocognitive tasks |
| Speed of Processing | Digit-Symbol Coding  Stroop Task – congruent condition  Trail Making Task  Verbal Fluency – Categories |
| Attention / Vigilance | Letter Cancellation |
| Working Memory | Corsi Tapping Blocks  Digit Span – Forward  Digit Span – Backwards  Letter-Number Span  Wechsler Memory Scale - Spatial Span |
| Verbal Learning and Memory | Hopkins Verbal Learning - Immediate Recall  Hong Kong List Learning Task – Acquisition  Hong Kong List Learning Task –Delayed  Rey Auditory Verbal Learning - Immediate Recall  Rey Auditory Verbal Learning - Delayed Recall |
| Visual Learning and Memory | Brief Visuospatial Memory Test—Revised |
| Reasoning and Problem Solving | Stroop Task – incongruent condition  Wisconsin Card Sorting Task  Neuropsychological Assessment Battery – Mazes |
| Social Cognition | TRENDS Facial Emotion Recognition  Mayer-Salovey-Caruso Emotional Intelligence - Managing Emotions |
| ^a^ Categorized according to the cognitive domains recommended by the MATRICS-NIHM Neurocognition Committee^26^ | |
