# Supplementary material for: Aerobic Exercise Improves Cognitive Functioning in People With Schizophrenia: A Systematic Review and Meta-Analysis
Source: Schizophr Bull. 2016 Aug 12;43(3):546–56. doi: 10.1093/schbul/sbw115 (PMC5464163; doi:10.1093/schbul/sbw115)

**Supplement 2.** Risk of bias summary: review authors' judgements about each risk of bias item for each included study.


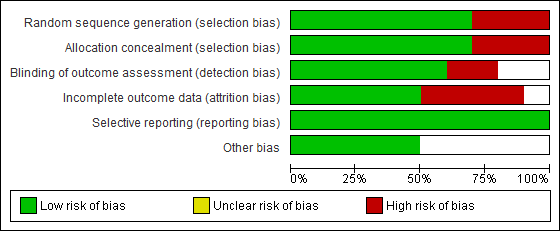


Risk of bias graph: review authors' judgements about each risk of bias item presented as percentages across all included studies.


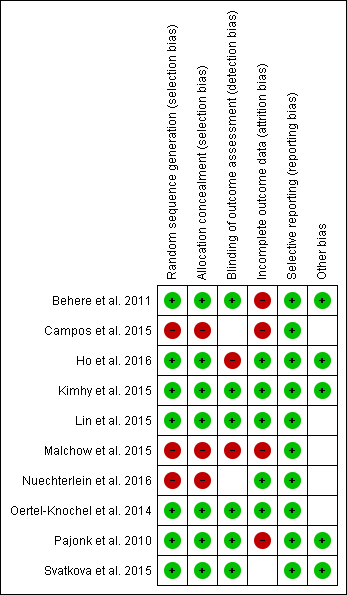

Supplement: Supplement_2._Risk_of_bias_assessments [file sbw115_suppl_Supplement_2._Risk_of_bias_assessments.docx]
