# Supplementary material for: Aerobic Exercise Improves Cognitive Functioning in People With Schizophrenia: A Systematic Review and Meta-Analysis
Source: Schizophr Bull. 2016 Aug 12;43(3):546–56. doi: 10.1093/schbul/sbw115 (PMC5464163; doi:10.1093/schbul/sbw115)
Supplement: Supplement_3._Meta_regression [file sbw115_suppl_Supplement_3._Meta_regression.docx]

Supplementary Figure 3a. Meta-regression of minutes per week of exercise and intervention effect size

Supplementary Figure 3b. Meta-regression of improvements in fitness and intervention effect size
